# Does the Consumer Sociodemographic Profile Influence the Perception of Aspects Related and Not Related to Food Safety? A Study in Traditional Spanish Street Markets

**DOI:** 10.3390/ijerph18189794

**Published:** 2021-09-17

**Authors:** Abel Verdú, Rafael Millán, Pedro Saavedra, Conrado Javier Carrascosa Iruzubieta, Esther Sanjuán

**Affiliations:** 1 Department of Animal Pathology and Production, Bromatology and Food Technology, Faculty of Veterinary, University of Las Palmas de Gran Canaria, 35001 Las Palmas de Gran Canaria, Spain; abel.verdusantana@gmail.com (A.V.); rafael.millan@ulpgc.es (R.M.); esther.sanjuan@ulpgc.es (E.S.); 2 Department of Mathematics, University of Las Palmas de Gran Canaria, 35001 Las Palmas de Gran Canaria, Spain; pedro.saavedra@ulpgc.es

**Keywords:** street markets, consumer profile, principal components, food safety, purchase criterion

## Abstract

Street food markets are important for local economic development, but they must also meet visitors’ demands while operating. Since consumers’ trust is based on their perception on different aspects of these markets, the aim of this work was to study which factors most affected their purchase decision criteria. A total of 950 surveys were collected in 21 street markets (Canary Islands, Spain), recording data from the consumers’ estimation on food safety-related items (i.e., hygiene conditions of market installations, products, and food handlers) as well as other categories (i.e., prices and staff professionalism). The gathered data let us determine whether sociodemographic consumers variables like age, gender, or education level influenced their perceptions. The scores showed a strong correlation, the subsequent principal components analysis explained 81% of variability only with the first two components. The level of tolerance toward all items underlies in the first component, which was significantly higher for those aged 60 and older, but no significant correlation was found for gender or level of education. The youngest participants were more demanding about food safety-related aspects, while the middle-aged group was more critical of prices. This was especially true of females, who demanded better quality:price ratios. Knowing these preferences could facilitate the development of more effective marketing strategies, helping make street markets more competitive.

## 1. Introduction

Regardless of whether street markets are of the farm market type or mobile street markets, they are important meeting points for producers and sellers of local food worldwide. At such markets, consumers can purchase products of plant and animal origin, as well as a wide array of the foods, beverages, and typical meals of the region [1].

Over the years, local authorities and traders have made efforts to improve these market installations. However, according to Lues et al. [2], certain elements that would ensure the food safety of the products on sale are still lacking. Permanent installations have better access to drinking water suitable for human use, lighting fixtures to improve arranged showcases, and the ability to store meals at suitable hot or cold temperatures [3]. Nonetheless, many installations are mobile and are assembled/disassembled at need. In these installations, the aforementioned elements are scarce or present shortcomings that might affect the salubriousness of the food stored and sold—thus implying higher risks to consumers [4].

Several studies have been conducted in this field to investigate the possible presence of pathogenic microorganisms in food and the possible risks to consumers when sanitary/hygienic conditions for the distribution, storage, preparation, exposure, or sale of food are unsuitable [5,6,7,8,9]. To this end, it has become essential to improve the conditions of these markets, in terms of their sales installations and food handlers, and to ensure compliance with safety measures.

Despite the aforementioned circumstances, street markets have prevailed for decades in our society. They are key to improving local economies in developing countries as places at which food is bought [10]. Most studies on this topic have centered on countries or regions in Africa and Asia, and comparatively few have focused on developed countries in the Americas or Europe [11]. General differences between both types of markets (i.e., farm and mobile) lie in their frequency of operation (daily vs. only one day/week), size (considerable vs. moderate), infrastructure, and safety/hygiene conditions (scarce vs. improved). Moreover, street markets in Asia and Africa act as essential hubs; they distribute food to consumers, aiding in commercial and economic development. This is in contrast to European and American street markets, which center on direct producer–consumer commerce (sometimes through an intermediary) and bring local producers to urban centers, favoring development in the rural areas of these regions by offering consumers quality local products [12,13].

For these traditional markets to survive, regardless of their location, consumer trust in street markets must develop; otherwise, producers will not have customers to continue their business. This trust is based on food quality-related aspects, consumers’ perception of adequate hygiene, or the influence of excellent prices as compared to other similar products [14,15,16]. Consumers have become increasingly aware of the hazards that they may face. However, efforts must be made to educate the general population to reduce food-related diseases [17,18].

Bearing in mind the importance of European-type street markets, which favor local economies and attempt to promote local products over supermarket offers, the present work used traditional street markets on the Canary Islands as a reference (Spain, Europe) to acquire data from the consumers who frequently buy from them. The overall objective was to record the estimation that consumers made of these markets’ food safety-related aspects (i.e., hygiene conditions of their installations, products and staff) and other factors not related to food safety (i.e., customer attention, services, and prices). This was intended to elucidate which criteria most affected their purchase decisions and to study whether they were influenced by sociodemographic consumer factors (i.e., age, gender, level of education). We also investigated the main reasons consumers buy food at street markets as opposed to shopping centers.

This information shed light on how important food safety and other aspects are to consumers, allowing producers to control those criteria and helping to make street markets more competitive. It also enabled us to measure consumers’ appreciation criteria according to their demographic profile characteristics, which could help to create more efficient marketing strategies with more specific approaches for each population sector. All this will help traditional markets continue to grow, generating more trust and increasingly more informed consumers who are concerned about their health and wellbeing.

## 2. Materials and Methods

### 2.1. Street Markets

**Figure 1:**
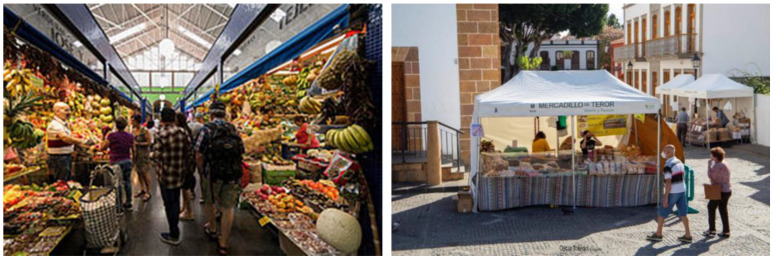
Street markets of Canary Islands.

This study was carried out in 21 traditional street markets on Gran Canaria Island (10) and Tenerife Island (11), which are the main islands of the Canaries (Spain). These markets were selected from approximately 40 currently operating on these two islands. They are considered the most important; more people go to them, both because local inhabitants and tourists perceive them as being traditional and because they all have stalls selling vegetables, dairy, and meat products. They also look homogeneous, with similar infrastructures, commercialization methods, and basic hygiene standards—which are backed by official health services. The number of visitors ranges between 1000 and 3000 people/day, which may vary according to their sizes or because of local festivities (i.e., more visitors when local festivities take place nearby). However, the number of visitors to each street market is relatively constant, except for when festivities occur. Figure 1 shows some examples of these street markets.

The markets are spread out all over these territories, both on the tourist coast and inland (where more local inhabitants visit them). They are classified by having fixed or mobile installations. Those located inside buildings, bays, or similar structures employ a prefabricated structure to house the different market stalls and are considered fixed installations. Mobile ones are arranged in town squares or on streets and are assembled and disassembled on market days.

### 2.2. Survey

At these markets, a survey was given to 950 consumers (475 on each island) who were randomly selected and surveyed in situ when they left the street market. The total size of the user population of the 21 markets was assumed to be about 50,000 people. In this scenario, a sample of 916 surveys would suffice to estimate any proportion with an error bound of 3.5% and 95% confidence.

A consumer was defined as anyone who had bought any product from any market stall on the survey day. They were asked if they were willing to be surveyed and participation was voluntary. The survey was conducted orally. The person conducting the survey asked consumers the questions and guided the interview if any question needed explaining. At the same time, the results of each survey were written down on paper under the supervision of the consumer to ensure that the data registered were correct (Supplementary Material). The survey taker ticked the selected options, and no open questions were included. Later, the information was keyed into a Microsoft Excel database to analyze them statistically. The surveys were conducted between April and May 2019 in the mornings and on different days of the week, because not all the street markets opened on the same days. Attempts were made to avoid going to these markets when festivities took place with a view to limit biases in the surveyed population and to include as many regular consumers as possible.

The surveys enabled us to assess the influence that the study items had on consumer purchase decisions. The survey was conducted as reported by Sánchez et al. [19], was modified based on other surveys conducted in similar works [20], and was verified by different professionals from the sector before it was conducted. All the questions asked are specified in Table 1, Table 2 and Table 3.

This survey included two parts. The first part was made up of demographic variables, including gender, age group (18–30 years, 31–59 years, >60), level of education (primary/secondary, vocational Training (VT)/higher secondary, university), and occupational status (student, worker, unemployed, pensioner). This allowed us to define consumer demands according to their profile and to orientate specific marketing actions on given population sectors.

The second part was employed to evaluate consumer perceptions of different market aspects. Its maximum score was 210 points for assessing some of the aspects linked with food safety, such as the products sold (50), food handlers’ training (40) and market hygiene (40), as well as others unrelated to food safety, such as professionalism (40) and price (30), using several questions about each item. The intention of this differentiation was to know which aspects consumers valued the most and whether they were related to food safety. One final question was asked, relating to consumers’ overall evaluation of the market in question (10). These questions were scored on a Likert scale, from 1 point, denoting “very bad/disagree” and 10 points, indicating “very good/agree”.

Finally, the surveyed people were asked about the main differences they observed between a street market and a shopping center. They were provided with six possible response options, but could only choose one (price, hygiene, freshness & quality, personal assistance, local products, or habit/proximity/convenience). Here, the intention was to elucidate which attributes consumers preferred when buying in traditional street markets rather than going to more modern shopping centers, and whether they were related to food safety (i.e., hygiene, freshness & quality) or other reasons. This could be important when emphasizing the permanence of such markets and when promoting them and ensuring their continuity.

### 2.3. Statistical Analysis

Categorical variables were expressed as frequencies and percentages, and continuous ones were expressed as medians and interquartile ranges (IQR = 25th–75th percentile). Percentages were compared, as appropriate, using either the Chi-square (χ^2^) or Fisher’ s exact test, and medians by the Kruskal–Wallis test. For the pairwise comparisons, the Tukey and Kramer (Nemenyi) test with Tukey–Dist approximation was employed for the independent samples [21].

Principal component analysis (PCA) is a widely used multivariate statistical technique to reduce the set of observed variables (i.e., items) to a smaller set of underlying variables (called principal components) based on patterns of correlation between the original variables [22]. These are obtained through the transformation of the observed variables into a set of linearly uncorrelated variables (see Manly, 1986 [23], for more details). The PCA has been previously used in assessment studies of the global food security index [24]. The consumer criteria herein analyzed were summarized in five items (products, prices, professionalism, food handlers’ training, and market hygiene), and they strongly correlated. Thus, we used the PCA to reduce them to two main components (PCs) to more clearly assess the associations between consumer criteria and the subjects’ characteristics (mainly age, gender and level of education).

Univariate analysis: Thus, the Pearson correlations between the observed variables were estimated. Principal components (PCs) were then obtained from the five observed items (totals) standardized to mean zero and variance one, which is denoted by $X=\left(X_{1},\ldots ,X_{5}\right)$. The first PC, $Y_{1}$, was defined as a linear combination of the X features in such a way that it captured its maximum variability ($Y_{1}=\overset{5}{\underset{i=1}{\sum }}\beta_{1,i}\times X_{i}$, being $\overset{5}{\underset{i=1}{\sum }}\beta_{1,i}^{2}=1$). Successive PCs, $Y_{2},Y_{3},Y_{4},Y_{5}$, were obtained in the same way, but were uncorrelated with the previous ones. It was proven that the variances of PCs satisfied $V\left(Y_{1}\right)>V\left(Y_{2}\right)>V\left(Y_{3}\right)>V\left(Y_{4}\right)>V\left(Y_{5}\right)$ and $\overset{5}{\underset{i=1}{\sum }}V\left(Y_{i}\right)=5$ (total variability). Therefore, the proportion of the total variability, explicated by PCs $Y_{j}$, was: ($V\left(Y_{j}\right)/\underset{i}{\sum }V\left(Y_{i}\right)$). The criterion on the number of main components to extract was based on the percentage of total variability that they explained. Note that the coefficients of the principal components were between −1 and 1.

Statistical significance was set at *p* < 0.05. Data were analyzed using R (R Foundation for Statistical Computing, Vienna, Austria).package, version 3.6.1 [25].

## 3. Results

Table 1 offers the sociodemographic characteristics of all those who took part in the survey and their evaluations, both globally and per gender. Data are expressed as frequencies and percentages for the sociodemographic variables, and as medians and interquartile ranges for the other evaluated items. The gender differences, and those according to levels of education, were significant (*p* = 0.006). Our data showed that the higher someone’ s level of education was, the more likely they would stop to answer, and the VT/higher secondary- and university-educated groups (especially females) were the most representative. The lower prices score for females was also significant as compared to males (*p* = 0.029), which demonstrated that women were disappointed with the fixed prices, demanding more quality when paying for a certain product.

The last question they were asked, which pertained to the differences they perceived between street markets and shopping centers, showed significant differences for both genders (*p* = 0.015). Females valued products’ freshness & quality, followed by their price. This order reversed for males. From all this, we determined that the females who went to these markets rather than to shopping centers prioritized the food safety-related aspects of products (freshness & quality), whereas males sought the best price. They all agreed that their purchase choice location was not related to habit/proximity/convenience.

Table 2 provides the obtained data on sociodemographic characteristics and evaluations according to age groups. For the participants’ characteristics, a significant difference was observed for age groups between the two islands, with more people with university education and workers in the 18–30 and 31–59 age groups, as well as pensioners who had completed VT/higher secondary education in the >60 age group.

The evaluation of those aspects surveyed in street markets by consumers showed that, for all the items, the >60 age group gave the highest scores, with the hygiene-related items included. This difference was significant compared to the other two age groups, except for market hygiene for the 18–30 age group and products and professionalism for the 31–59 age group. These data may have revealed the influence of age on the way consumers perceive services and give scores in surveys.

The answers for the last question (on differences between street markets and shopping centers) were significantly different for our three age groups (*p* = 0.049). The first option, selected by the 18–30 and 31–59 age groups, was product freshness & quality, followed by price. However, this order reversed for the >60 age group. From this, we noted that the consumers aged 59 years and younger who visited these markets instead of shopping centers expected to find advantages in food safety-related aspects of products (freshness & quality), while those aged 60 and older sought the best price. The fact that young people attached little importance to local products stood out. Once again, habit/proximity/convenience had the least influence for all three age groups.

Table 3 shows the sociodemographic characteristics and evaluations according to the participants’ level of education. Females who completed university education formed the significant majority on Gran Canaria, while males who had completed primary/secondary education were the majority on Tenerife. Most were in the 31–59 age group and worked.

For the items that assessed the street market, no significant differences were detected among the participants according to the three levels of education considered, except for structure cleanliness (included in market hygiene (*p* = 0.033)), a food safety-related factor that was better evaluated by the primary/secondary level—who came across as less critical. Nor did their level of education significantly affect the different reasons they gave for choosing to buy in street markets rather than shopping centers.

**Figure 2:**
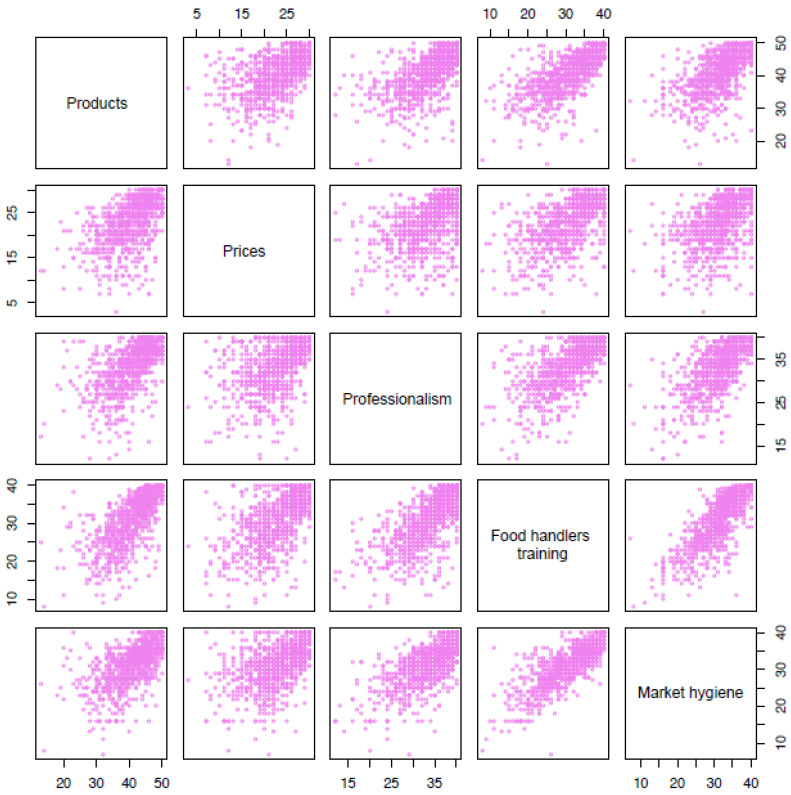
Dispersion diagram for all the pairs of the total five analyzed items.

Table 4 shows the Pearson correlation matrix (*p*-value) corresponding to the five considered items. Figure 2 displays the pairwise cloud of points for the same variables. Note that all the correlations exceed 0.5.

As we can see, higher Pearson correlation (0.802; *p* < 0.001) was found in the scores given for the items about food handlers training, as perceived by the participants, and the degree of market hygiene. Conversely, the lowest level of association (0.499; *p* < 0.001) appeared between the professionalism level for attending customers and product prices (data offered in Figure 2 are widely dispersed). Note that the matrix is symmetric (cor(*X_i_*,*X_j_*) = cor(*X_j_*,*X_i_*)) and, consequently, each correlation is only showed once (triangular matrix).

Thus, the high correlations between the observed variables suggested transforming them into a smaller number of variables using the principal components. This allowed us, to analyze the associations between age, gender, and level of education of consumers with their assessments of the markets, in addition to identifying the underlying patterns of the variables.

Table 5 shows the five PC deduced from the five analyzed items. Each PC is a linear combination of the standardized original variables. The variances of PCs are shown in Table 6. The first PC explained 70.2% of the total variability of the original data, while the first two PCs together accounted for 81.2% (Table 6). Therefore, we reduced the five variables to these two PCs, as follows:

*Y*_1_ = 0.4411 × X_1_ + 0.3978 × X_2_ + 0.4417 × X_3_ + 0.4872 × X_4_ + 0.4633 × X_5_ (1)

*Y*_2_ = −0.0749 × X_1_ − 0.8532 × X_2_ + 0.3572 × X_3_ + 0.0988 × X_4_ + 0.3594 × X_5_ (2)

where $X_{1},X_{2},X_{3},X_{4},X_{5}$ are the standardized original items.

The coefficients expressed the contributions that each variable made to the PC (Table 5). Note that all the coefficients in the first PC were similar, with values between 0.39 and 0.49 (by construction, coefficients of the PCs were quantities between −1 and 1). Food handlers’ training contributed the most and prices the least.

Note that the first PC increased with the five variables, while the second decreased with prices and products and increased with professionalism, food handlers’ training and market hygiene. Therefore, high values in the first PC corresponded to giving the five variables a higher score, while high values in the second PC corresponded to high scores in professionalism and hygiene aspects, like food handlers’ training and market hygiene, and low in products and prices. However, the contributions of products (−0.0749) and food handlers’ training (0.0988) were low. Thus, people who give high values in the second PC evaluated the professionalism and hygiene aspects well, but the prices badly.

The total variance of the five standardized variables was 5, while the variance of the first PC was 3.509 (the 70.2% of the total). As a result, this first PC explained 70.2% of the total variability of data (Table 6). The second PC was uncorrelated with the first (no overlapping information) and explained 11.1% of the total variability. Therefore, both PCs explained 81.2% of the total variability of data. This allowed us to describe a dataset of five variables by means of the two principal components.

Table 7 summarizes the two first PCs according to age groups, gender and level of education. Multiple comparisons were made by the Tukey–Kramer–Nemenyi test.

Both PCs showed significant differences between age groups. The first component underlaid the level of tolerance toward all items and was significantly higher for the >60 group, but not significant for gender or level of education. The second PC indicated significant differences between genders, but not for any component according to level of education.

**Figure 3:**
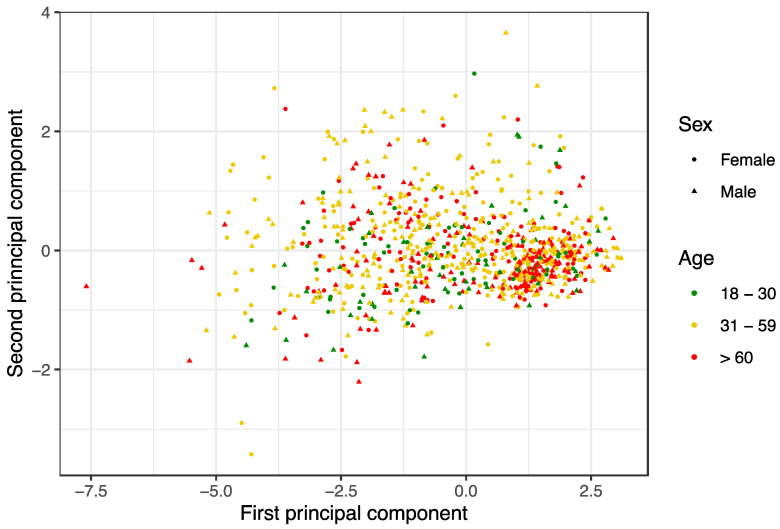
Scatter plot for the two first principal components according to age group and gender. The first principal component explains 70.2% of data variability, and both together account for 81.2%. Note that the consumers aged 60 years and older tend to give high values on the first PC. The very high values of the second PC correspond to consumers who have given high scores for professionalism and market hygiene (i.e., low demand for these aspects) but low values to the prices (i.e., disagreement with the prices).

As significance differences occurred mainly in accordance with age groups and gender, Figure 3 depicts the scatter plot for the first two PCs.

It was noteworthy that the >60 years age group tended to evaluate the five totals as important (and was less critical), as reflected by the fact that the median of the first PC was significantly higher in this group (1.070 as opposed to −0.138 in the 18–30 age group and 0.134 in the 31–59 age group) (Table 7). It was also noteworthy that the two first age groups did not significantly differ for the first PC. To understand what this meant, it was necessary to consider the second PC.

The second PC showed no significant difference between 18–30 and >60 age groups (Table 7). However, the first PC indicated differences, which meant that the 18–30 age group tended to value hygiene-related aspects less, namely, food handlers’ training and market hygiene (the items with higher coefficients on the first PC; Table 5) because this group was more demanding. The second PC was significantly lower in the youngest age group, as compared to the 31–59 age group (Table 7), which means that they were indeed more inclined to give a lower score for hygiene-related items and were less demanding about prices and products (negative coefficients in the second PC; Table 5).

Table 7 shows that, for both PCs, the 31–59 age group tended to be less critical about hygiene-related aspects than the 18–30 age group. 

Regarding gender differences, for the second PC, Figure 3 and Table 7 show a higher value for females, which implies that they were stricter with prices and products (negative coefficients on the second PC; Table 5) and less strict with staff professionalism and other food safety-related aspects like market hygiene and food handlers’ training (positive coefficients on the second PC; Table 5).

## 4. Discussion

The results of this study coincided with the work by Sook Theng et al. [26], who found different consumer purchase intentions in accordance with how they perceived good practices displayed by the food handlers who worked nights on food stalls in Malaysia. The consumers perceived hygiene as better when more knowledge and good practices were applied. [27,28,29]. This also came across in the present study; however, this perception was linked with consumer age, as our youngest age group showed the most interest in hygiene practices.

Just as the evaluation made by our consumers between market food handlers’ training and market hygiene correlated well, other studies also detected a relationship between a stall’ s general hygiene status and the hygiene conditions of the production, handling, and distribution of the food sold by the handling staff [30,31], as well as its microbiological quality [32]. Ghartey and Antwi [33] also stressed that food handlers in south Ghana with a higher level of education washed their hands better.

Both locations and types of market installations have been highlighted in several studies as being particularly interesting [34,35,36]. The present study found that the type of market installation had no influence on conditioning consumer preferences. Participants did not contemplate location in their purchase choice because they lived on islands where very long distances did not come into play and had a wide range of similar street markets. Consumers opted for other factors when choosing between shopping in one place or another (mainly product freshness & quality, and price). Other studies found that these factors could include the type of products, which are usually local [37,38], or other factors like convenience (closeness to their home, transport connections, etc.), crowds, or treatment received, among others [39,40]. Mascarello et al. [41] divided consumer profiles into two large groups: people who made their decisions according to food organoleptic factors and those who valued the place or preparation methods. Another relevant point to be considered is product traceability [42], or the information accompanying the product regarding its origin, production characteristics or the usage of phytosanitary products or veterinary medicines. More and more, these factors are appreciated and valued by consumers.

Liu and Niyongira [43] found that, in China, females, families with children and older adults were among those who possessed more knowledge and voiced more concern when purchasing food. The higher their level of education, the more that perception held true. Bil Der et al. [44] detected significant gender differences in Turkish consumers, and differences according to the city they came from or their level of education when making purchase decisions. In our case, clear differences were found for age, but variance between genders were not as evident (only for prices) or level of education (only for structure cleanliness).

As in the study of Nagoya et al. [45], most of our participants were satisfied with the market hygiene level, product availability, and quality. This revealed that consumers perceived the efforts made by health authorities and market workers to improve food safety and sale conditions. Notwithstanding, these efforts must continue to improve consumer trust in street markets and similar places worldwide [46,47,48]. Consumer training in food safety must continue [49,50] in order to end the circular seller-consumer cycle, which provides self-feedback, and to involve everyone in food safety-related activities [51].

## 5. Conclusions

The choice of consumers to shop at street markets instead of going to supermarkets was based mainly on their perception of product freshness & quality (stressed by those aged 59 years or younger and by females) and prices (stressed by those aged 60 years or older and by males). When we requested consumer participation in situ, we noted that the majority who stopped to answer the survey had a purchasing power basis (workers or pensioners) and a higher level of education. However, their level of education did not influence their perception of the aspects evaluated in the survey.

After visiting street markets, with these evaluations, they were able to recognize the marked association between food safety-related aspects (e.g., market hygiene vs. food handlers’ training). In fact, the scores of all the aspects related to attention and food quality/safety were significantly related, with major differences for age groups. The youngest participants were less rigorous about prices and products (probably because of their lack of experience in buying food from these markets) but were more demanding about hygiene-related aspects. The 31–59 age group was more critical about the quality-related aspects of products and their prices, probably because they are responsible for their family economy. Those in the >60 age group were the least demanding about all the evaluated items, whether they were related to food safety and not. This finding reflected their willingness to visit these traditional markets. As for gender influence, females were stricter about prices and products than about professionals’ correct treatment or other food safety-related aspects (market hygiene and food handlers’ training), which indicated that females, especially those in the 31–59 age group, demanded a better quality:price ratio.

Once the most valued aspects in each consumer sector—which impact their purchase decisions at markets—are determined according to their gender and age groups, it will be easier to apply strategies that cover their food safety demands or other aspects that could help markets to remain commercially competitive. In this way, each market and each stand within the market will be able to decide how to make purchases more attractive to its consumers. Those most visited by the young public should invest in improving the hygienic conditions of their facilities and their food handlers. On the other hand, other markets, preferred by middle-aged and female populations, may have to focus their sales strategy toward offering quality products at competitive prices.

## Figures and Tables

**Table 1:** Global and per gender analysis.

	Global *n* = 950	Female *n* = 523	Male *n* = 427	*p*-Value
AGE (years)				0.053 †
18–30	121 (12.7)	75 (14.3)	46 (10.8)	
31–59	508 (53.5)	287 (54.9)	221 (51.8)	
>60	321 (33.8)	161 (30.8)	160 (37.5)	
ISLAND				0.948 †
Gran Canaria	475 (50.0)	261 (49.9)	214 (50.1)	
Tenerife	475 (50.0)	262 (50.1)	213 (49.9)	
LEVEL OF EDUCATION/*n* = 600				0.006 †
Primary/Secondary	184 (30.7)	89 (25.8)	95 (37.3)	
VT/Higher Secondary	207 (34.5)	122 (35.4)	85 (33.3)	
University	209 (34.8)	134 (38.8)	75 (29.4)	
OCCUPATIONAL STATUS/*n* = 600				0.179 †
Student	44 (7.3)	26 (7.5)	18 (7.1)	
Worker	362 (60.3)	214 (62.0)	148 (58.0)	
Unemployed	97 (16.2)	59 (17.1)	38 (14.9)	
Pensioner	97 (16.2)	46 (13.3)	51 (20.0)	
FACILITIES				0.795 †
Fixed	890 (93.7)	489 (93.5)	401 (93.9)	
Mobile	60 (6.3)	34 (6.5)	26 (6.1)	
PRODUCTS (50 *)	42 (37–45)	42 (37–45)	42 (37–45)	0.433 ‡
Quality	9 (8–10)	9 (8–9)	9 (8–10)	0.067 ‡
Labelling/Information	8 (6–9)	8 (6–9)	8 (6–9)	0.472 ‡
Variety	8 (7–9)	8 (7–9)	8 (7–9)	0.956 ‡
Local/Artisanal	9 (8–10)	9 (8–10)	9 (8–10)	0.407 ‡
Freshness	9 (8–10)	9 (8–10)	9 (8–10)	0.319 ‡
PRICES (30 *)	24 (20–27)	24 (20–27)	25 (20–28)	0.029 ‡
Quality/Cost ratio	9 (7–10)	9 (7–9)	9 (7–10)	0.215 ‡
Price choice	8 (6–9)	8 (6–9)	8 (6–9)	0.108 ‡
Higher price—better quality association	8 (7–9)	8 (6–9)	8 (7–9)	0.052 ‡
PROFESSIONALISM (40 *)	35 (30–37)	35 (30–37)	35 (30–38)	0.510 ‡
Received treatment	9 (8–10)	9 (8–10)	9 (8–10)	0.621 ‡
Waiting time	9 (7–9)	9 (7–9)	9 (7–9)	0.496 ‡
Information/Advice	9 (7–9)	9 (7–9)	9 (7–9)	0.965 ‡
Looking after customers	9 (8–10)	9 (8–10)	9 (8–10)	0.314 ‡
FOOD HANDLERS’ TRAINING (40 *)	33 (27–36)	33 (27–36)	32 (26–36)	0.753 ‡
Presentation of products	8 (7–9)	8 (7–9)	8 (7–9)	0.393 ‡
Food handling	9 (7–9)	8 (7–9)	9 (7–9)	0.757 ‡
Correct uniform	8 (6–9)	8 (6–9)	8 (6–9)	0.276 ‡
Uniform hygiene	9 (6–9)	9 (7–9)	8 (6–9)	0.424 ‡
MARKET HYGIENE (40 *)	32 (28–35)	32 (29–35)	32 (28–36)	0.950 ‡
Table cleanliness	8 (7–9)	8 (7–9)	8 (7–9)	0.790 ‡
Equipment cleanliness	8 (7–9)	8 (7–9)	8 (7–9)	0.573 ‡
Structure cleanliness	8 (7–9)	8 (7–9)	8 (7–9)	0.872 ‡
General hygiene	9 (7–9)	9 (7–9)	9 (7–9)	0.974 ‡
OVERALL EVALUATION (10 *)	9 (7–9)	9 (7–9)	9 (7–9)	0.651 ‡
TOTAL (210 *)	174 (151–188)	174 (152–187)	174 (150–189)	0.491 ‡
MARKET/SHOPPING CENTRE DIFFERENCE				0.015 †
Price	282 (29.8)	139 (26.6)	143 (33.6)	
Hygiene	70 (7.4)	40 (7.7)	30 (7.1)	
Freshness & Quality	333 (35.2)	209 (40.0)	124 (29.2)	
Personal assistance	113 (11.9)	59 (11.3)	54 (12.7)	
Local products	124 (13.1)	64 (12.3)	60 (14.1)	
Habit/Proximity/Convenience	25 (2.6)	11 (2.1)	14 (3.3)	

Data are frequencies (%) and medians (IQR); (†) Chi-square test; * Maximum score of the evaluated item; (‡) Kruskal–Wallis test.

**Table 2:** Analysis of consumer age groups.

	18–30 *n* = 121	31–59 *n* = 508	>60 *n* = 321	*p*-Value
GENDER				0.053 †
Female	75 (62.0)	287 (56.5)	161 (50.2)	
Male	46 (38.0)	221 (43.5)	160 (49.8)	
ISLAND				<0.001 †
Gran Canaria	51 (42.1)	289 (56.9)	135 (42.1)	
Tenerife	70 (57.9)	219 (43.1)	186 (57.9)	
LEVEL OF EDUCATION/*n* = 600				0.003 †
Primary/Secondary	31 (31.3)	96 (27.2)	57 (38.5)	
VT/Higher Secondary	29 (29.3)	119 (33.7)	59 (39.9)	
University	39 (39.4)	138 (39.1)	32 (21.6)	
OCCUPATIONAL STATUS/*n* = 600				<0.001 †
Student	34 (34.3)	9 (2.5)	1 (0.7)	
Worker	54 (54.5)	250 (70.8)	58 (39.2)	
Unemployed	10 (10.1)	75 (21.2)	12 (8.1)	
Pensioner	1 (1.0)	19 (5.4)	77 (52.0)	
FACILITIES				0.050 †
Fixed	110 (90.9)	471 (92.7)	309 (96.3)	
Mobile	11 (9.1)	37 (7.3)	12 (3.7)	
PRODUCTS (50 *)	40 (36–44) ^a^	42 (37–45) ^a,b^	43 (38–45) ^b^	0.016 ‡
Quality	9 (7–9)	9 (8–9)	9 (8–10)	0.048 ‡
Labelling/Information	7 (6–8)	8 (6–9)	8 (7–9)	<0.001 ‡
Variety	8 (7–9)	8 (7–9)	8 (7–9)	0.685 ‡
Local/Artisanal	9 (7–9)	9 (8–10)	9 (8–10)	0.120 ‡
Freshness	9 (8–10)	9 (8–10)	9 (8–10)	0.006 ‡
PRICES (30*)	24 (20–26) ^a^	23 (19–27) ^a^	26 (22–28) ^b^	<0.001 ‡
Quality/Cost ratio	9 (7–9)	8 (7–9)	9 (8–10)	<0.001 ‡
Price choice	8 (7–9)	8 (6–9)	8 (7–9)	<0.001 ‡
Higher price—better quality association	8 (6–9)	8 (6–9)	9 (7–10)	<0.001 ‡
PROFESSIONALISM (40 *)	33 (28–37) ^a^	35 (30–37) ^a,b^	36 (32–38^) b^	0.003 ‡
Received treatment	9 (7–10)	9 (8–10)	9 (8–10)	0.003 ‡
Waiting time	8 (7–9)	9 (7–9)	9 (8–9)	<0.001 ‡
Information/Advice	8 (7–9)	8 (7–9)	9 (8–9)	0.081 ‡
Looking after customers	8 (7–10)	9 (8–10)	9 (8–10)	0.037 ‡
FOOD HANDLERS’ TRAINING (40 *)	31 (26–35) ^a^	32 (26–36) ^a^	34 (29–36) ^b^	0.001 ‡
Presentation of products	8 (7–9)	8 (7–9)	9 (7–9)	0.014 ‡
Food handling	8 (6–9)	8 (7–9)	9 (8–9)	<0.001 ‡
Correct uniform	8 (6–9)	8 (5–9)	8 (6–9)	0.034 ‡
Uniform hygiene	8 (6–9)	8 (6–9)	9 (7–9)	<0.001 ‡
MARKET HYGIENE (40 *)	32 (28–35) ^a,b^	32 (28–35) ^a^	33 (30–36) ^b^	0.013 ‡
Table cleanliness	8 (6–9)	8 (7–9)	9 (8–9)	0.004 ‡
Equipment cleanliness	8 (7–9)	8 (7–9)	8 (7–9)	0.153 ‡
Structure cleanliness	8 (7–9)	8 (7–9)	8 (7–9)	0.153 ‡
General hygiene	8 (7–9)	8 (7–9)	9 (8–9)	<0.001 ‡
OVERALL EVALUATION (10 *)	8 (7–9)	8 (7–9)	9 (8–9)	<0.001 ‡
TOTAL (210 *)	166 (146–185)	170 (149–187)	183 (155–190)	<0.001 ‡
MARKET/SHOPPING CENTRE DIFFERENCE				0.049 †
Price	41 (33.9)	138 (27.2)	103 (32.3)	
Hygiene	11 (9.1)	37 (7.3)	22 (6.9)	
Freshness & Quality	45 (37.2)	194 (38.3)	94 (29.5)	
Personal assistance	12 (9.9)	56 (11.0)	45 (14.1)	
Local products	12 (9.9)	71 (14.0)	41 (12.9)	
Habit/Proximity/Convenience	0	11 (2.2)	14 (4.4)	

Data are frequencies (%) and medians (IQR; (†) Chi-square test; * Maximum score of the evaluated item; (‡) Kruskal–Wallis test; ^a,b^ Different letters indicate significant differences (*p* < 0.05) according to the Tukey–Kramer–Nemenyi post hoc test. Totals only.

**Table 3:** Analysis of consumer levels of education.

	Primary/Secondary *n* = 184	VT/Higher Sec. *n* = 207	University *n* = 209	*p*-Value
GENDER				0.006 †
Female	89 (48.4)	122 (58.9)	134 (64.1)	
Male	95 (51.6)	85 (41.1)	75 (35.9)	
AGE (years)				0.003 †
18–30	31 (16.8)	29 (14.0)	39 (18.7)	
31–59	96 (52.2)	119 (57.5)	138 (66.0)	
>60	57 (31.0)	59 (28.5)	32 (15.3)	
ISLAND				0.008 †
Gran Canaria	80 (43.5)	98 (47.3)	122 (58.4)	
Tenerife	104 (56.5)	109 (52.7)	87 (41.6)	
OCCUPATIONAL STATUS/*n* = 600				0.049 †
Student	14 (7.6)	13 (6.3)	17 (8.1)	
Worker	96 (52.2)	128 (61.8)	138 (66.0)	
Unemployed	33 (17.9)	38 (18.4)	26 (12.4)	
Pensioner	41 (22.3)	28 (13.5)	28 (13.4)	
FACILITIES				0.276 †
Fixed	170 (92.4)	187 (90.3)	183 (87.6)	
Mobile	14 (7.6)	20 (9.7)	26 (12.4)	
PRODUCTS (50 *)	38 (34–43)	38 (34–43)	39 (36–43)	0.445 ‡
Quality	8 (7–9)	8 (7–9)	8 (7–9)	0.164 ‡
Labelling/Information	7 (6–8)	7 (6–8)	7 (6–8)	0.500 ‡
Variety	8 (6–9)	7 (6–9)	8 (7–9)	0.428 ‡
Local/Artisanal	8 (7–9)	8 (7–9)	8 (7–9)	0.383 ‡
Freshness	8 (7–9)	8 (7–9)	9 (7–9)	0.375 ‡
PRICES (30 *)	21 (18–24)	21 (18–24)	21 (17–24)	0.919 ‡
Quality/Cost ratio	7 (6–8)	8 (6–9)	8 (6–9)	0.502 ‡
Price choice	7 (6–8)	7 (5–8)	7 (5–8)	0.675 ‡
Higher price—better quality association	7 (6–8)	7 (5–8)	7 (5–8)	0.864 ‡
PROFESSIONALISM (40 *)	32 (28–36)	32 (28–35)	32 (28–35)	0.652 ‡
Received treatment	8 (7–10)	8 (7–9)	9 (7–9)	0.556 ‡
Waiting time	8 (7–9)	8 (7–9)	8 (7–9)	0.847 ‡
Information/Advice	8 (6–9)	8 (6–9)	8 (7–9)	0.813 ‡
Looking after customers	8 (7–10)	8 (7–9)	8 (7–9)	0.171 ‡
FOOD HANDLERS’ TRAINING (40 *)	29 (25–33)	28 (24–32)	28 (23–32)	0.208 ‡
Presentation of products	8 (7–9)	8 (7–9)	8 (7–9)	0.127 ‡
Food handling	8 (6–9)	7 (6–9)	7 (6–8)	0.293 ‡
Correct uniform	7 (6–8)	7 (5–8)	7 (5–8)	0.285 ‡
Uniform hygiene	7 (6–9)	7 (5–8)	7 (5–8)	0.413 ‡
MARKET HYGIENE (40 *)	31 (27–34)	30 (26–33)	29 (26–33)	0.168 ‡
Table cleanliness	8 (7–9)	7 (6–9)	8 (6–9)	0.080 ‡
Equipment cleanliness	8 (7–9)	8 (7–9)	8 (6–8)	0.066 ‡
Structure cleanliness	8 (6–9)	7 (6–8)	7 (6–8)	0.033 ‡
General hygiene	8 (6–9)	7 (6–9)	7 (6–8)	0.604 ‡
OVERALL EVALUATION (10 *)	8 (7–9)	8 (7–9)	8 (7–9)	0.851 ‡
TOTAL (210 *)	157 (142–171)	154 (141–170)	157 (140–170)	0.616 ‡
MARKET/SHOPPING CENTRE DIFFERENCE				0.089 †
Price	53 (29.1)	63 (30.6)	51 (24.4)	
Hygiene	20 (11.0)	30 (14.6)	20 (9.6)	
Freshness & Quality	68 (37.4)	85 (41.3)	91 (43.5)	
Personal assistance	22 (12.1)	17 (8.3)	17 (8.1)	
Local products	17 (9.3)	9 (4.4)	27 (12.9)	
Habit/Proximity/Convenience	2 (1.1)	2 (1.0)	3 (1.4)	

Data are frequencies (%) and medians (IQR); (†) Chi-square test; * Maximum score of the evaluated item; (‡) Kruskal–Wallis test.

**Table 4:** Pearson correlations (*p*-value).

	Products	Prices	Professionalism	Food Handlers’ Training
Prices	0.549 (<0.001)			
Professionalism	0.602 (<0.001)	0.499 (<0.001)		
Food handlers’ training	0.700 (<0.001)	0.611 (<0.001)	0.677 (<0.001)	
Market hygiene	0.607 (<0.001)	0.512 (<0.001)	0.679 (<0.001)	0.802 (<0.001)

**Table 5:** Coefficients of the principal components (PC).

Totals	First	Second	Third	Fourth	Fifth
Products	0.4411	−0.0749	−0.8726	−0.0641	−0.185
Prices	0.3978	−0.8532	0.3053	−0.0813	−0.1183
Professionalism	0.4417	0.3572	0.2373	−0.7856	0.06145
Food handlers’ training	0.4872	0.0988	0.0452	0.3929	0.7723
Market hygiene	0.4633	0.3594	0.2948	0.4666	−0.5929

**Table 6:** Analysis of the dimension of the feature vector.

	Variance	% of Variance	% of Accumulated Variance
First	3.509	70.173	70.173
Second	0.553	11.054	81.227
Third	0.403	8.054	89.282
Fourth	0.359	7.179	96.460
Fifth	0.177	3.540	100.000

**Table 7:** Associations between the characteristics of consumers and PCs.

	First PC	*p*-Value	Second PC	*p*-Value
AGE (years)		<0.001 †		<0.001 †
18–30	−0.138 (−1.641; 1.267) ^a^		−0.134 (−0.612; 0.173) ^a^	
31–59	0.134 (−1.419; 1.359) ^a^		−0.025 (−0.386; 0.440) ^b^	
>60	1.070 (−0.958; 1.623) ^b^		−0.172 (−0.462; 0.165) ^a^	
GENDER		0.472 †		0.006 †
Female	0.374 (−1.288; 1.400)		−0.069 (−0.385; 0.395)	
Male	0.386 (−1.360; 1.530)		−0.140 (−0.485; 0.235)	
LEVEL OF EDUCATION		0.597 †		0.830 †
Primary/Secondary	−0.867 (−1.945; 0.325)		0.117 (−0.363; 0.600)	
VT/Higher Secondary	−1.061 (−2.068; 0.159)		0.000 (−0.427; 0.585)	
University	−0.827 (−2.134; 0.110)		0.114 (−0.481; 0.633)	

Data are medians (IQR); Different superscripts indicate significant differences (*p* < 0.05) according to the Tukey–Kramer–Nemenyi post hoc test; (†) Kruskal–Wallis test.

## Data Availability

Not applicable.

## Supplementary Materials

The following are available online at https://www.mdpi.com/article/10.3390/ijerph18189794/s1.
