# Supplementary material for: Does the Consumer Sociodemographic Profile Influence the Perception of Aspects Related and Not Related to Food Safety? A Study in Traditional Spanish Street Markets
Source: Int J Environ Res Public Health. 2021 Sep 17;18(18):9794. doi: 10.3390/ijerph18189794 (PMC8470163; doi:10.3390/ijerph18189794)
Supplement: Supplementary file 1 [file ijerph-18-09794-s001.zip › ijerph-1339815-supplementary.pdf]

SURVEY OF HYGIENIC-SANITARY CONDITIONS AND CONSUMER SATISFACTION IN TRADITIONAL STREET MARKTS					
Please, mark your group of sociodemographic profile in each item.					
1	Gender	○ Male		○ Female	
2	Age (years)	○ 18 - 30	○ 31 - 59	○ > 60	
3	Level of education	○ First / Secondary	○ VT / Higher Sec.	○ University	
4	Occupational Status	○ Student	○ Worker	○ Unemployed	○ Pensioner

Please, rate each item in order to evaluate this Traditional Street Market (1 point is very bad / disagree, 10 points is very good / agree)			
		SCORE	***MAX. SCORE***
**5**	**Products**	**-**	**50**
**5.1.**	Quality of the products		10
**5.2.**	Labelling / Information of the products		10
**5.3.**	Variety of products		10
**5.4.**	Local / Artisan products		10
**5.5.**	Freshness of the products		10
**6**	**Prices**	**-**	**30**
**6.1.**	Quality / Cost Ratio		10
**6.2.**	You choose food depending on the price		10
**6.3.**	You associate higher prices to higher quality		10
**7**	**Professionalism / Customer care**	**-**	**40**
**7.1.**	Received treatment from the staff		10
**7.2.**	Waiting time		10
**7.3.**	Information / Advice during shopping		10
**7.4.**	The staff is looking after customers		10
**8**	**Food Handlers Training**	**-**	**40**
**8.1.**	Presentation of the products		10
**8.2.**	Propper food handling by the staff		10
**8.3.**	Staff wears correct uniform		10
**8.4.**	The staff uniform hygiene is correct		10
**9**	**Higiene del mercado**	**-**	**40**
**9.1.**	Table, chairs and other surfaces cleanliness		10
**9.2.**	Equipment cleanliness (displays, exhibitors)		10
**9.3.**	Structure cleanliness (floors, ceilings, stalls…)		10
**9.4.**	General hygiene of the market		10
**10**	**Overall evaluation**	**-**	**10**
**10.1.**	Satisfaction general level	10	10
**Total score**		**-**	**210**

**11.**	***Please, mark the main difference that you found between a traditional street market and a shopping centre.***	
**11.1.**	Price	
**11.2.**	Hygine	
**11.3.**	Freshness and quality	
**11.4.**	Personal Assistance	
**11.5.**	Local products	
**11.6.**	Habit/ Proximity/ Convenience	

Many thanks for your collaboration
